# Cohabitation and unmet care needs in later life

**DOI:** 10.1093/geronb/gbag136

**Published:** 2026-07-11

**Authors:** Sarah E Patterson, Matthew R Wright, Vicki A Freedman

**Affiliations:** Institute for Social Research, University of Michigan, Ann Arbor, Michigan, United States; Department of Sociology, Appalachian State University, Boone, North Carolina, United States; Institute for Social Research, University of Michigan, Ann Arbor, Michigan, United States

**Keywords:** Cohabitations, Marriage, Caregiving

## Abstract

**Objectives:**

More older Americans are cohabiting with a partner. However, it is unclear whether cohabitation serves as an alternative to marriage for meeting daily needs in later life. This study examines whether receipt of partner assistance and experience of unmet care needs differ by union status and gender.

**Methods:**

Data are from the 2012, 2016, and 2023 National Health and Aging Trends Study, including 4,283 community-dwelling older adults (aged 66+; 4,070 married, 213 cohabiting) who coreside with an opposite-sex partner and are in need of care. Outcomes include receipt of health-related assistance “care,” receipt of non-health-related assistance “help,” overall assistance hours, and unmet care needs. We estimate logistic and linear regression models by union status and assess differences for men vs. women.

**Results:**

In adjusted models, cohabitation is not associated with receipt of care or help but is associated with fewer hours of help (−24.13; *p* < .05). However, cohabitation associations differ by gender (*p* < .05) for unmet care needs, with greater risks conferred by cohabitation for women but not men.

**Discussion:**

Cohabitation functions similarly to marriage among older adults who are in need of care, for both care and help received, but cohabitors receive fewer hours than spouses from their partners. Cohabiting women experience more adverse consequences related to unmet needs than married women, with a larger gender gap in unmet care needs among cohabiting couples than among married couples. Continued tracking of the impact of cohabitation as the U.S. population continues to age is warranted.

An increasing share of the older adult population in the United States is forming nonmarital partnerships, including cohabiting relationships (i.e., living with a partner but remaining unmarried). In 2022, among adults aged 50 and older, 13% of men and 8% of women were in cohabiting relationships (Marino, 2022). With the overall aging of the U.S. population, increasing rates of gray divorce (Brown & Lin, 2012), and declining remarriage rates (Raley & Sweeney, 2020), there are now more older adults eligible to cohabit (Carr & Utz, 2020; Cooney & Dunne, 2001). Cohabitation rates have also increased in part because of more supportive attitudes toward cohabitation among cohorts entering later life (Brown & Wright, 2016). Because activity limitations increase with age (Freedman & Spillman, 2014), a growing share of those cohabiting in later life may need assistance with daily activities. Romantic partners are often the primary source of assistance for partnered older adults with limitations (Lin, 2024). But whether cohabitation operates equivalently to marriage in later life with respect to partner support is less clear.

Some scholars have framed cohabitation as an “incomplete” institution in the United States, in which cohabiting couples do not have the same degree of established norms as married couples and therefore have to construct and negotiate dynamics that work for their relationships (Nock, 1995). This incompleteness could potentially influence exchange patterns within families (Patterson, 2023), including assistance that is both related to health and functioning “care” and unrelated to health and functioning “help.” This incompleteness may be especially influential for women who may prefer cohabitation to marriage (Kgadima & Leburu, 2022; McWilliams & Barrett, 2014; Perelli-Harris et al., 2014; Perelli-Harris & Bernardi, 2015; Watson & Stelle, 2011). Other scholars have suggested that cohabitation operates as an alternative form of marriage in the second half of the life course (Brown & Wright, 2017; Julian & Brown, 2025; King & Scott, 2005). Evidence for this perspective, which emphasizes that cohabiting older adults behave in ways that are similar to married older adults, has been found across several facets of later life relationships, including income pooling among those aged 60–65 (Brown & Wright, 2017; Wright, 2020; Wright et al., 2023).

To date, only one published study has investigated care received in later-life cohabiting relationships in the United States. Among a sample of older adults with care needs (i.e., those reporting difficulty with any activity), cohabitors were less likely than married individuals to receive care from their partners (Noël-Miller, 2011). However, the data used by Noël-Miller were from 2000 to 2006, preceding the broader acceptance of cohabitation in the older adult population. Limitations in the data also precluded distinguishing care received related to health and functioning from help with household activities (e.g., doing the laundry, shopping) that is commonplace when couples live together. Moreover, the analysis did not explore differences by gender even though cohabitation patterns (Marino, 2022), reasons for preferring cohabitation (King & Scott, 2005; McWilliams & Barrett, 2014; Talbott, 1998; Watson & Stelle, 2011), and care patterns (Dukhovnov & Zagheni, 2015; Glauber, 2017) differ for men and women. In short, we lack a good understanding of caregiving and help received from a partner in later life, and of whether older men and women who cohabit interact in ways similar to married partners.

Using pooled data from the 2012, 2016, and 2023 National Health and Aging Trends Study (NHATS), we investigate receipt of partner care and partner help, total hours of assistance (either care or help) received from all caregivers and the partner, and the experience of adverse consequences related to unmet care needs among married and cohabiting older adults aged 66 and older who reside in the community with their opposite-sex partner and who are in need of care. We investigate competing hypotheses about cohabitation as an incomplete institution (Nock, 1995) and cohabitation as an alternative to marriage (Brown & Wright, 2017; King & Scott, 2005). We hypothesize that if cohabitation is an incomplete institution, then older adults who cohabit with a partner but are not married to them may be less likely than married individuals to receive care and help; conversely, if cohabitation serves as an alternative to marriage, we should see similar levels of care and help for the two groups. We also expect that the relationship between cohabiting status and partner assistance will vary by gender, with women experiencing no difference by union status and a larger gender gap between men and women among cohabiting couples relative to married couples. Taken together, this study provides new insights into caregiving received in later-life cohabiting relationships, as well as the meaning of these nonmarital unions for older men and women.

## Background

Cohabitation has become more prevalent among older adults over the past two decades. In 2020, nearly one in four cohabiting couples in the United States had at least one partner aged 50 or older (U.S. Census Bureau, 2020), but fewer studies have looked at older adults aged 65 and older in particular. Partnerships in older adulthood are important for many outcomes, including mental and physical health as well as mortality (Carr & Utz, 2020; Liu & Reczek, 2012; Patterson et al., 2020; Umberson & Karas Montez, 2010). Partners are especially important in older adulthood in relation to care receipt (Lin, 2024), the need for which increases with age, with the onset of activity limitations (Freedman & Spillman, 2014). Therefore, there is potentially a sizeable and growing number of older adults cohabiting for whom partner support may be important to understand.

### Cohabitation as an incomplete institution

Theoretically, cohabitation has been viewed as an incomplete institution in the United States, in which relationships lack norms and expectations (Nock, 1995), leading to lower rates of exchange in families (Noël-Miller, 2011; Patterson, 2023). The expectations for a spouse to provide care when needed are normative as a part of marital responsibility or devotion (Cash et al., 2019), but expectations within cohabitation may be less clear. Cohabiting partners may therefore have to negotiate and construct their own dynamics that best fit their relationships because pre-determined “scripts” are not necessarily available to them. Due to this flexibility, older adults may prefer cohabitation to remarriage because they are better able to maintain their independence (De Jong Gierveld, 2004; King & Scott, 2005). For some, then, cohabitation may be a way to enjoy companionship and avoid caregiving obligations that are traditionally expected with marriage (McWilliams & Barrett, 2014; Talbott, 1998; Watson & Stelle, 2011), whereby cohabiting older adults are then less likely to receive assistance from their partner.

Other research on cohabiting couples and various forms of family negotiations reinforces the notion of incompleteness. For instance, a recent study found that U.S. midlife cohabiting couples (ages 50–65) were less likely to pool their incomes than their remarried counterparts, though older cohabiting couples (ages 60–65) were not (Wright et al., 2023). Likewise, a study of Finnish older adults found that cohabitation was less stable than marriage among older adults aged 65 and older (Moustgaard & Martikainen, 2009). In this paradigm, cohabiting partners may be less likely than their married counterparts to provide care because of the freedom that cohabitation affords couples (Noël-Miller, 2011).

### Cohabitation as an alternative to marriage

An alternative viewpoint posits that as cohabitation diffuses through the population (Liefbroer & Dourleijn, 2006) and becomes more acceptable at older ages (Brown & Wright, 2016), it may become more institutionalized and thus function similarly to marriage among contemporary cohorts of older adults (Brown & Wright, 2017; King & Scott, 2005). Whereas cohabiting relationships earlier in the life course tend to be short-lived, cohabitations in the second half of the life course are longer-lasting and more stable (Brown et al., 2012). These unions are unlikely to lead to marriage or separation, but instead most commonly end in the death of a partner (Brown et al., 2012).

Mounting evidence suggests that cohabitation functions as an alternative to marriage among older adults (Brown & Wright, 2017; Julian & Brown, 2025; King & Scott, 2005). For instance, married and cohabiting older adults report similar levels of relationship quality (Brown & Kawamura, 2010; Wright, 2020). Compared to married individuals, cohabiting older adults also tend to be similar in their reports of emotional satisfaction, demands, and time spent together (Brown & Kawamura, 2010). Cohabiting and married partners in the United States also have similar risk for health declines in later life, for instance, in the likelihood of developing cognitive impairment (Liu et al., 2019). Likewise, the psychological well-being of older adults who cohabit with their partner, measured across several dimensions, is on par with that of their married counterparts (Wright & Brown, 2017). Therefore, if cohabitation operates as an alternative to marriage in later life, cohabiting relationships and marriages may function similarly with regard to caregiving received.

### Studies on older adults, cohabitation, and care

To date, few studies of care received in later life have compared cohabiting older adults with their married counterparts. Moreover, existing studies examining caregiving among older cohabiting and married couples typically focus on younger-older adults (ages 50–65) rather than those aged 65 and older, or within other national contexts, finding few differences (e.g., the Netherlands, see van Groenou, 2019). Yet, commentaries on the emergence of cohabitation have questioned whether unmarried partners will provide “comparable care” to married spouses (Manning & Brown, 2015, p. 96), necessitating an exploration of these trends among older adults.

To our knowledge, only one published study has investigated caregiving and care receipt among middle-aged and older (aged 51 and older) cohabiting adults in the United States (Noël-Miller, 2011), and none have investigated unmet care needs. Noël-Miller (2011) found, in a sample with care needs, that cohabiting partners were less likely than married spouses to receive care from a partner with daily activities. However, the summary measure used in this study did not distinguish between receipt of care related to health and functioning and non-health-related help with these tasks. Although assistance with activities like bathing or mobility can be assumed to be due to health limitations, the motivation for carrying out household and administrative tasks is not always health-related. Instead, household help may reflect couples’ decisions about the division of labor within their household, which may be patterned by gender (Sheehan & Tucker-Drob, 2019). Yet, studies on late-life care rarely distinguish differences between care and help. This begs the question whether care functions similarly among cohabiting and married unions while household help functions differently, as cohabiting couples seek to preserve their independence.

Noël-Miller’s (2011) study found no difference in total hours of assistance received by cohabitation status but a larger share of their overall care from a cohabiting partner compared to married partners after adjusting for other characteristics. However, data for this study were from 2000 to 2006, prior to the rise in later-life cohabitation occurring over the past decade (Stepler, 2017). That study also included adults aged 51 and older, ages during which partner-related care patterns change rapidly (Dukhovnov & Zagheni, 2015) and did not focus on ages at highest risk for care (i.e., the average age in their study was 65). Moreover, unmet care needs were not examined, despite the high prevalence among older adults generally (Freedman & Spillman, 2014) and especially among those with high need (Beach & Schulz, 2017). In addition, differences in outcomes by gender were not explored. Thus, it is unclear whether Noël-Miller’s (2011) findings hold broadly in contemporary society among older married and cohabiting men and women.

### Differences between men and women

Older men are more likely to cohabit than older women (Marino, 2022). Men are also, on average, less likely to provide care or help to a partner compared to women (Allen, 1994; Dukhovnov & Zagheni, 2015; Glauber, 2017). Recent evidence suggests that women in opposite-sex partnerships continue to provide high levels of partner support over time, but the rate of men providing care to their partner has nearly doubled from about 6% of men aged 51 and older in 2002 to about 11% in 2018 (Cha & Ailshire, 2026). Despite the increase, men providing partner care are more likely to perform low-intensity and secondary care tasks, compared to women who are providing intensive, “primary” care tasks (Cha & Ailshire, 2026). This trend suggests a possible narrowing of the strong gendered expectations with respect to household activities that have been previously identified (Sheehan & Tucker-Drob, 2019).

Yet, it is not clear whether there are disparate implications of cohabitation for men and women. Compared with older men, older women may be more drawn to the incomplete nature of cohabitation and its dual benefits of companionship and independence (King & Scott, 2005; Talbott, 1998). Older women may also prefer cohabitation to marriage because it allows for greater flexibility and is not necessarily predicated on gendered norms often attached to marriage (Kgadima & Leburu, 2022; McWilliams & Barrett, 2014; Perelli-Harris et al., 2014; Perelli-Harris & Bernardi, 2015; Watson & Stelle, 2011). Gender norms around caregiving are also pervasive, despite growing gender egalitarianism among recent cohorts (Brooks & Bolzendahl, 2004; Pessin, 2018). Further, theoretical frameworks around men and care have emphasized “specification of tasks,” in that it is normative for men and women to provide different forms of care within families (Finley, 1989). Other scholars of men’s care have echoed the importance of understanding men’s care through more managerial, technical, or skill-based tasks rather than traditional care tasks (Russell, 2001), with men being more likely to help with household tasks than personal care (Zhang et al., 2025). However, some scholars have noted the lack of ability to investigate these trends further due to both theoretical and data limitations of measures (Freedman et al., 2024), and other work has shown that married men are more likely than married women to attribute household help received as a continuation of prior division of labor before the onset of health conditions (Allen et al., 1993). Therefore, we may expect no differences by cohabitation status, but emphasize the importance of distinguishing between care and help activities.

## Data and methods

To explore differences between cohabiting and married couples with respect to care, help, and unmet care needs, we used the 2012, 2016, and 2023 (Rounds 2, 6, and 13) NHATS. NHATS is a longitudinal national panel study that is representative of the U.S. Medicare population aged 65 and older. The 2012, 2016, and 2023 samples represent adults aged 66 and older. NHATS is sponsored by the National Institute on Aging (grant number NIA U01AG32947) and is conducted by Johns Hopkins University (Freedman et al., 2026).

When pooled in this way, NHATS provides a recent, large, and representative sample of the 66 and older population sampled at three time points over the decade. We limit our sample to older adults who reside in the community (i.e., not in a residential community or facility of any kind) with an opposite-sex partner and are either married to or cohabiting with that partner. We also limit the sample to those older adults “in need” of care (i.e., they report either receiving help or having difficulty with any household, self-care, or mobility activity; a comparable sample to Noël-Miller, 2011). In doing so, we rely on the “Other Person” reports in NHATS to identify coresidential partners, including coresidential boyfriends/girlfriends. Missingness is generally low in NHATS (e.g., 1% missing for race/ethnicity), so we used mean/mode imputation for the limited number of missing cases in order to retain the full sample (a listwise deletion sample yielded the same results). Our final analytic sample comprised *N *= 4,283 older adults (4,070 Married; 213 Cohabiting).

### Measures

#### Union status

We measure union status as either married to an opposite-sex partner or cohabiting (i.e., living with a partner but unmarried) with an opposite-sex partner or boyfriend/girlfriend. Opposite-sex partners are identified through a question about changes in marital status since the last round; boyfriends/girlfriends are identified through questions about the relationship of individuals who reside in the household to the sample person.

#### Care, help, total assistance hours, and adverse consequences from unmet care needs

Although the literature is inconsistent in how it defines caregiving (see Freedman & Wolff, 2020, for a recent discussion), the NHATS framework and survey design allow researchers to distinguish care that is related to an older adult’s health or functioning from help that occurs for other reasons (Freedman et al., 2026). This approach is particularly relevant for household activities such as doing laundry or preparing meals, which may be done by one partner for another or by partners together for reasons unrelated to health or functioning. We therefore adopt the NHATS framework approach to classifying assistance with self-care, mobility, or household activities for health or functioning reasons as “care” and assistance with household activities for other reasons as “help.”

To construct a measure of care received from a partner, we first identified NHATS participants receiving assistance in the last month with self-care activities (bathing, dressing, eating, and toileting), mobility activities (getting out of bed, getting around inside, and getting outside) or household activities (laundry, shopping, making hot meals, managing money, and managing medications) for health or functioning reasons. For each activity, respondents were asked to report who provided care and, if not already ascertained, the relationship of the helper to the NHATS respondent. We used this additional information from the NHATS Other Person file to identify those receiving care from a coresidential partner (including coresidential spouse, unmarried, coresidential partner, or coresidential boyfriend/girlfriend).

To construct a measure of help received from a partner, we first identified participants who had household activities carried out with them or for them by another person in the last month only for reasons other than health or functioning or who had someone assist with administrative or medical tasks (handling other money matters, sitting in on doctor visits, handling insurance matters, driving most) in the last year. We then limited this indicator to those receiving this help from a partner.

To construct hours of assistance received in the last month, we drew information from the Other Person file. For each person providing help, the NHATS respondent reports hours of help received in the last month (not specifying whether that help is related to health and functioning). Following Freedman et al. (2014), we calculate hours of assistance in the last month for each helper based on a series of questions about each helper’s schedule, the number of days per week or month, and the number of hours per day on days when assistance was provided. We then sum across individuals providing assistance to create total hours of assistance received as well as the proportion received from the partner (out of all hours received). We include the share of hours of assistance received from the partner to be able to compare to Noël-Miller’s (2011) findings.

For adverse consequences reflecting unmet care needs (Allen & Mor, 1997), respondents were asked the following question(s) if they reported receiving help, having difficulty, or not doing the activity in the last month: “In the last month, did you ever go without [consequence] because it was too difficult to do by yourself/no one was there to help or do that for you?” Consequences included: self-care (e.g., going without a shower/bath, not getting dressed, going without eating, wetting or soiling clothing), mobility (e.g., having to stay in bed, having to stay inside, and not being able to going outside), or household chores conditional on health and functioning (e.g., go without clean laundry, groceries/personal items, a hot meal, handling bills/banking, missed medicines).

#### Control variables

We include information about the older adult and their partnership. We include the number of years in the relationship; this information is only asked in the initial round, so we bring forward information from the round the respondent entered (e.g., for 2023 Samples Person who entered in Round 1, we add 12 years). For a spouse/partner whose relationship began after the older adult’s initial round, we count the number of rounds that the partner has been in the Other Person file. We also include whether their partner is older, whether their partner needs help with personal care (i.e., eating, bathing, dressing), whether they talk to their partner about important matters, and logged total couple income from the closest round of collection (using imputations provided by NHATS; Hu & Freedman, 2024) adjusted to 2022 dollars. Moreover, we account for the number of children (biological, stepchildren), age (65–74, 75–84, 85+), whether the older adult is female, primary race/ethnicity (derived variable in NHATS; non-Hispanic White, non-Hispanic Black, Hispanic, non-Hispanic Other), and whether the older adult has a college degree. We also control for two factors that reflect the underlying physical and cognitive capacity of the individual. Physical capacity was self-reported as being able to complete a series of low- or high-difficulty tasks (walking, stairs, weight, flexibility, reach, and grip) following Kasper et al. (2017). Responses are coded 0 if the respondent is not able to do the tasks, 1 if they are able to complete a less difficult version, and 2 if they can complete the more difficult version. The score is the sum of all scores on the 6 items. We also control for whether the respondent has either possible or probable dementia (using the NHATS coding scheme; Kasper et al., 2013). We code whether the sample person had a proxy respondent. Although not common to have a proxy respondent, about two-thirds of proxy respondents were partners (*n *= 227/335); see the sensitivity analysis section.

### Analytic plan

First, we provide descriptive statistics for older adults who are in need of care overall and by union status. Second, overall and by union status, we estimate care patterns for four outcomes: received health-related care from their partner; received non-health-related help from their partner; total partner assistance hours received (if any); and whether the older adult experienced any adverse consequences related to unmet care needs. Third, we estimate logistic regression models for the four care outcomes by union status, and test for differences by gender using an interaction term between cohabitation and gender. We present a figure with predictive margins for any significant interactions (with the corresponding regression in Supplementary Material). Estimates are weighted and adjusted for survey design and repeated observations. All analyses were completed in Stata 19.0.

## Results

Most coresidential opposite-sex partnered older adults aged 66 and older who are in need of care are married (95.0%); only 5.0% are cohabiting (Table 1). The proportion of older adults cohabiting did not change appreciably over the 11-year time frame (4.4% in 2012 vs. 5.5% in 2023; n.s.). Characteristics of married and cohabiting older adults are quite similar across most partnership and demographic characteristics, with a few exceptions. Married older adults are more likely than those cohabiting to have spent more years together (43.5 vs. 18.2 years; *p* < .001), have a higher average couple income ($81,299 vs. 64,785; *p* < .001), have more children on average (3.0 vs. 2.3; *p* < .001), have a college degree (30.3% vs. 21.8%; *p* < .05), and have a proxy respondent (6.2% vs. 1.4%; *p* < .001).

**Table 1:** Weighted descriptive statistics of characteristics of older adults (aged 66 and older) in need of care who coreside with their opposite-sex partner in the community, means (#) or percentages (%).

Variables	Older adults in need of care			
	All	Married	Cohabiting	Sig. diff.
***N***	4,283	4,070	213	
**Weighted % of the sample**	—	95.0	5.0	
**Partnership characteristics**				
Years married or living together (#)	42.2	43.5	18.2	***
Partner is older (%)	34.8	35.2	26.8	
Partner needs help with personal care (%)	9.5	9.5	8.0	
Talk to partner about important matters (%)	76.6	76.8	72.1	
Total household income ($; in 2022 dollars)	80,467	81,299	64,785	***
**Older adult characteristics**				
Physical capacity score (#)	8.2	8.2	8.3	
Dementia (possible or probable) (%)	19.9	20.1	15.6	
Number of children (#)	2.9	3.0	2.3	***
Age (%)				
65–74	50.7	50.4	56.7	
75–84	39.4	39.7	34.0	
85+	9.9	9.9	9.3	
Female (%)	44.1	44.3	39.4	
Race/ethnicity (%)				
White, non-Hispanic	80.6	81.1	72.8	
Black, non-Hispanic	6.6	6.5	9.4	
Hispanic	8.0	7.8	11.7	
Other, non-Hispanic	4.8	4.7	6.2	
College degree (%)	29.9	30.3	21.8	*
Has a proxy survey respondent (%)	6.0	6.2	1.4	***

*Note*. Sig. Diff. = significant difference between married and cohabiting older adults. Data are pooled from the 2012, 2016, and 2023 National Health and Aging Trends Study (NHATS). Estimates are weighted and adjusted for survey design and repeated observations.

* *p* < .05.

*** *p* < .001.

Focusing on unadjusted bivariate outcomes (see Table 2), among older adults in need of care, the weighted percentage receiving care from a partner is similar for married and cohabiting older adults (45.2% vs. 41.6%; n.s.), as is the proportion receiving help (89.1% vs. 88.7%; n.s.). Overall, there are no significant differences between married and cohabiting older adults in the percentage receiving care or help with specific domains of tasks (e.g., self-care, mobility, household care, household help, administrative tasks). However, significant differences emerge in the number (but not percentage) of hours of assistance in the last month from a partner (97.4 for married vs. 69.9 for cohabiting; *p* < .01) and hours of assistance from anyone (118.6 vs. 85.1; *p* < .001). Despite these differences, the percentages of married and cohabiting older adults experiencing adverse consequences due to unmet care needs are similar (30.7% vs. 27.6%; n.s.).

**Table 2:** Weighted descriptive statistics of help and care received by older adults (aged 66 and older) in need of care who coreside with their opposite-sex partner in the community.

Variables	All	Married	Cohabiting	Sig. diff.
**Care from partner**				
Received care	45.0	45.2	41.6	
Self-care (eating, getting cleaned up, using the toilet, getting dressed)	25.9	26.2	20.2	
Mobility (getting in and out of bed, getting around inside, getting around outside)	19.0	19.1	16.4	
Household care (due to health/functioning; laundry, meals, shopping, banking, help with medicine)	30.8	30.9	29.2	
**Help from partner**				
Received help	89.1	89.1	88.7	
Household help (for non-health reasons; laundry, meals, shopping, banking, help with medicine)	76.1	76.2	73.0	
Administrative tasks (handling other money matters, sitting in on doctor visits, handling insurance matters, driving most or other)	66.1	66.4	61.5	
**Hours of assistance (Care or Help) received from partner, if any care/help received from partner**				
Partner hours	96.0	97.4	69.9	**
Total hours (from anyone)	116.9	118.6	85.1	**
Percentage of hours received from partner	84.3	84.2	85.7	
**Adverse consequences related to unmet care needs**				
Adverse consequence for any activity	27.8	27.6	30.7	
Self-care (eating, getting cleaned up, using the toilet, getting dressed)	10.5	10.6	9.9	
Mobility (getting in and out of bed, getting around inside, getting around outside)	15.8	15.6	19.6	
Household help (due to health/functioning; laundry, meals, shopping, banking, help with medicine)	11.9	12.0	11.5	

*Note*. Sig. Diff. = significant difference between married and cohabiting older adults. *N *= 4,283 (4,070 married; 213 cohabiting); sample sizes for hours of assistance sub-analyses: *N *= 3,872 (3,683 Married; 189 Cohabiting). Data are pooled from the 2012, 2016, and 2023 National Health and Aging Trends Study (NHATS). Estimates are weighted and adjusted for survey design and repeated observations.

** *p* < .01.

In the main effect model controlling for partnership and demographic characteristics (Table 3), the receipt of care from a partner does not vary by union status among older adults in need of care (−0.09; n.s.). However, women are more likely to receive care from their male partner (0.26; *p* < .05) than men are to receive care from their female partner, when controlling for both partner and older adult characteristics (see sensitivity section for additional analysis). The interaction term between cohabiting and female is not significant, meaning the effect of cohabiting does not vary for older men and women with care needs. Findings for help are similar: the receipt of help from a partner does not differ by union status (Table 3, −0.07; n.s.); women are less likely to receive help from their partner than men, but the interaction term is not significant.

**Table 3:** Regression coefficients for logistic regression models predicting care and help received from partner by older adults (aged 66 and older) in need of care who coreside in the community.

Variables	Received care from partner (logit)				Received help from partner (logit)			
	Coeff.	*SE*	Coeff.	*SE*	Coeff.	*SE*	Coeff.	*SE*
**Cohabiting (vs. Married)**	−0.09	0.23	−0.12	0.28	−0.07	0.31	−0.07	0.40
**Female (vs. Male)**	0.26*	0.11	0.26*	0.12	−0.96***	0.16	−0.96***	0.16
× Cohabiting			0.07	0.43			−0.01	0.54
**Partnership characteristics**								
Years married or living together	−0.00	0.00	−0.00	0.00	−0.00	0.01	−0.00	0.00
Partner is older	−0.04	0.12	−0.04	0.12	−0.21	0.15	−0.21	0.15
Partner needs help with personal care	−1.02***	0.18	−1.02***	0.18	−1.81***	0.15	−1.81***	0.15
Talk to partner about important matters	0.46***	0.12	0.46***	0.12	1.31***	0.15	1.31***	0.15
Total household income (logged)	−0.02	0.07	−0.02	0.07	0.12	0.11	0.12	0.11
**Older adult characteristics**								
Physical capacity score	−0.31***	0.02	−0.31***	0.02	−0.02	0.02	−0.02	0.02
Dementia (possible or probable)	0.61***	0.13	0.61***	0.13	0.13	0.17	0.13	0.17
Number of children	0.01	0.03	0.01	0.03	−0.01	0.03	−0.01	0.03
Age (65–74 omitted)								
75–84	0.09	0.10	0.09	0.10	−0.16	0.16	−0.16	0.16
85+	0.09	0.14	0.09	0.14	−0.65**	0.21	−0.65**	0.21
Race (White, non-Hispanic omitted)								
Black, non-Hispanic	−0.33*	0.14	−0.33*	0.14	−0.87***	0.17	−0.87***	0.17
Hispanic	−0.44**	0.17	−0.44**	0.17	−0.76***	0.21	−0.76***	0.22
Other, non-Hispanic	−0.50	0.29	−0.50	0.29	−0.59	0.33	−0.59	0.33
College degree	0.03	0.11	0.03	0.11	0.25	0.17	0.25	0.17
Has a proxy respondent	0.78**	0.24	0.78**	0.24	0.24	0.25	0.24	0.25
**Survey year (2012 omitted)**								
2016	0.23*	0.11	0.23*	0.11	0.20	0.15	0.20	0.15
2023	0.09	0.11	0.08	0.11	−0.04	0.16	−0.04	0.16
**Constant**	2.04**	0.79	2.04**	0.79	1.45	1.18	1.45	1.18

*Note*. *N *= 4,283 (4,070 Married; 213 Cohabiting). Data are from the 2012, 2016, and 2023 National Health and Aging Trends Study (NHATS). Estimates are weighted and adjusted for survey design and repeated observations.

* *p* < .05.

** *p* < .01.

*** *p* < .001.

Several partnership and older adult characteristics are associated with care received from a partner (Table 3). Having a partner who needs help with personal care, having better physical function (e.g., a greater physical capacity score), and being Black, non-Hispanic, or Hispanic (relative to White, non-Hispanic) are each associated with being less likely to receive care from a partner. Talking to their partner about important matters, having dementia, or having a proxy respondent is associated with a greater likelihood of receiving care from a partner. Except for dementia and proxy respondent, these covariates are similarly associated with receipt of help, as is being aged 85 or older. The association for receiving more care is significantly higher in 2016 than in 2012.

Turning to hours of assistance received from a partner (Table 4), the main effect model shows that cohabiting older adults receive fewer hours of assistance than married older adults (−24.13; *p* < .05). Women receive fewer hours of assistance from their partner compared to men in the main effect model (−52.85; *p* < .001), but the interaction term suggests no difference in the effect of union status between women and men (2.95; n.s.). Findings for the percentage of hours of assistance from partner differ from those for total hours: cohabiting status is not significant (0.00; n.s.); women receive a smaller percentage of their total assistance hours from their partner compared to men (−0.07; *p* < .001); and the interaction term is not significant.

**Table 4:** Regression coefficients for hours of assistance from partner and percent of assistance hours by partner for older adults (aged 66 and older) in need of care who coreside in the community.

Variables	Total hours of assistance from partner, if any (OLS)				Percent of hours of assistance from partner, if any (OLS)			
	Coeff.	*SE*	Coeff.	*SE*	Coeff.	*SE*	Coeff.	*SE*
**Cohabiting (vs. Married)**	−24.13*	9.50	−25.24*	10.87	0.00	0.02	0.01	0.03
**Female (vs. Male)**	−52.85***	6.40	−53.02***	6.66	−0.07***	0.01	−0.07***	0.01
× Cohabiting			2.95	16.32			−0.02	0.04
**Partnership characteristics**								
Years married or living together	−0.09	0.19	−0.09	0.19	−0.00	0.00	−0.00	0.00
Partner is older	11.08	6.60	11.13	6.62	0.01	0.01	0.01	0.01
Partner needs help with personal care	−50.49***	8.49	−50.49***	8.49	−0.23***	0.03	−0.23***	0.03
Talk to partner about important matters	16.46*	6.72	16.47*	6.72	0.06***	0.01	0.06***	0.01
Total household income (logged; 2022 dollars)	−1.99	4.15	−1.98	4.15	−0.00	0.01	−0.00	0.01
**Older adult characteristics**								
Physical capacity score	−9.40***	0.99	−9.40***	0.99	0.01***	0.00	0.01***	0.00
Dementia (possible or probable)	43.55***	9.43	43.56***	9.44	−0.01	0.01	−0.01	0.01
Number of children	−2.44	1.59	−2.44	1.59	−0.01*	0.00	−0.01*	0.00
Age (65–74 omitted)								
75–84	17.44**	6.13	17.41**	6.15	0.02	0.01	0.02	0.01
85+	19.94*	9.89	19.90*	9.90	−0.06***	0.02	−0.06***	0.02
Race (White, non-Hispanic omitted)								
Black, non-Hispanic	26.00*	10.13	26.01*	10.13	−0.05***	0.01	−0.05***	0.01
Hispanic	35.85**	13.39	35.83**	13.39	−0.05**	0.02	−0.05**	0.02
Other, non-Hispanic	−0.51	13.05	−0.58	13.08	0.01	0.02	0.01	0.02
College degree	−10.33	5.84	−10.34	5.84	−0.00	0.01	−0.00	0.01
Has a proxy respondent	99.38***	18.43	99.38***	18.43	0.01	0.02	0.01	0.02
**Survey year (2012 omitted)**								
2016	−20.20**	7.02	−20.21**	7.02	−0.03**	0.01	−0.03**	0.01
2023	−31.42***	7.05	−31.43***	7.05	−0.01	0.01	−0.01	0.01
**Constant**	211.42***	46.77	211.43***	46.77	0.85***	0.09	0.85***	0.09

*Note*. Coeff. = coefficient; OLS = ordinary least squares. *N *= 3,872 (3,683 Married; 189 Cohabiting). Data are from the 2012, 2016, and 2023 National Health and Aging Trends Study (NHATS). Estimates are weighted and adjusted for survey design and repeated observations.

* *p* < .05.

** *p* < .01.

*** *p* < .001.

Several other factors are associated with partner assistance hours and the percentage of assistance hours (Table 4). If their partner needs help with personal care or if the respondent has a higher physical capacity score, the respondent receives fewer hours from their partner. However, if the older adult confides in their partner, has dementia, is 75–84 years old, is Black, non-Hispanic, or Hispanic, or has a proxy respondent, they receive more hours from their partner. The number of hours of assistance received from a partner also declined over the period. Factors associated with a lower percentage of assistance hours received from the partner include if the partner needs help with personal care, the number of children, being 85 years or older, and being Black, non-Hispanic, or Hispanic. Confiding in the partner or having a higher physical capacity score is associated with a higher percentage.

**Figure 1:**
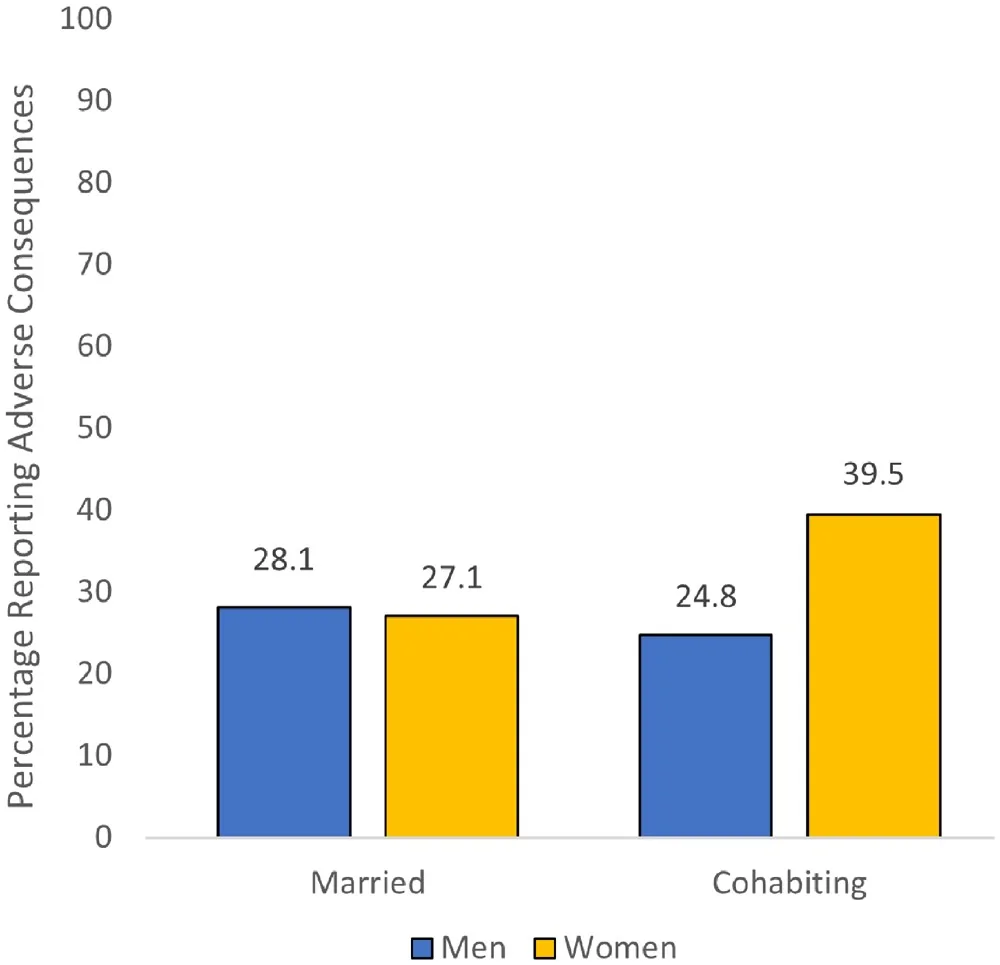
Predictive margins for adverse consequences among older adults (aged 66 and older) in need of care who coreside with their opposite-sex partner in the community, by union status and gender. *N* = 3,872 (3,683 Married; 189 Cohabiting). Data are pooled from the 2012, 2016, and 2023 National Health and Aging Trends Study (NHATS). Estimates are weighted and adjusted for survey design and repeated observations. *p < .05. **p <. 01. ***p < .001.

Focusing on adverse consequences (Supplementary Table 1), the main effect model suggests no difference between cohabiting and married older adults (0.19; n.s.), nor a difference between men and women (−0.00; n.s.). The interaction model does suggest some differences. Linear combinations of parameters tests show no significant effect of union status among men (−0.22; n.s.), but the effect among women is significant (*p* < .05). Moreover, the cohabiting effect for men and women differ from each other (as evidenced by the significant interaction term; 0.91; *p* < .05). The model reveals no gender gap in married couples (−0.06; n.s.), and additional lincom tests show that the gender gap among cohabiting couples, with cohabiting men being less likely to have unmet care needs compared to cohabiting women, is significant (*p* < .05). Other characteristics associated with unmet care needs include having a partner who needs help with personal care, having dementia, and having a college degree, but having a higher physical capacity score, being aged 85 and older, or being Black, non-Hispanic, are associated with being less likely to have unmet needs among partnered older adults in need of care. Unmet needs among older adults in need of care are higher in 2023 compared to 2012. Figure 1 illustrates predictive margins from Supplementary Table 1 (controlling for partner and older adult characteristics). Over one-third (39.5%) of cohabiting women report an adverse consequence compared to one-quarter (24.8%) of cohabiting men, 27.1% of married women, and 28.1% of married men.

### Sensitivity analysis

Additional estimates are provided for illustrative purposes. Among partnered men in need of care, there are only a few significant differences between those who are married and cohabiting (Supplementary Table 2). Married men have lived with their partner for more years (41.9 vs. 18.3; *p* < .001), have more children (3.0 vs. 2.3; *p* < .01), are older (*p* < .05), are more likely to have a college degree (35.1% vs. 22.2%; *p* < .05), and have a proxy respondent (7.2% vs. 1.3%; *p* < .01). Among partnered women in need of care, married women are more likely to have more years together (45.5 vs. 18.1; *p* < .001), have an older partner (63.4% vs. 38.2%; *p* < .01), have more children (2.9 vs. 2.3; *p* < .05), and have a proxy respondent (4.9% vs. 1.5%; *p* < .05). Additional tests between cohabiting women and men show that cohabiting women are more likely to have an older partner (38.2% vs. 19.4%; *p* < .05) but are less likely to talk to their partner about important matters (60.4% vs. 79.7%; *p* < .05) and have a lower physical capacity score (7.1 vs. 9.2; *p* < .01).

Models estimating unadjusted bivariate estimates for individual types of care or help show few differences among men and among women, but some differences when comparing cohabiting men and women (Supplementary Table 3). Among men, cohabiting men are receiving fewer hours of assistance from their partner (*p* < .01) as well as overall total hours (*p* < .001) compared to married men; they also have lower levels of unmet self-care needs (4.7% vs. 10.0%; *p* < .05). Among women, cohabiting women are more likely to have unmet needs (45.4% vs. 29.3%; *p* < .05), especially mobility unmet care needs (31.1% vs. 18.2%; *p* < .05) compared to married women.

Additional adjusted regression models for individual types of care, help, or unmet care needs show no significant interactions (potentially due to small sample sizes), but some trends vary by gender (Supplementary Tables 4 and 5). Women are less likely than men to receive self-care care (*p* < .001) from their partner, but more likely than men to receive mobility care (*p* < .001) and health-related household care (*p* < .001) (Supplementary Table 4). Among non-health-related tasks, women are less likely to receive household help (*p* < .001) but more likely to receive administrative help (*p* < .01) from their partner compared to men. No significant gender or cohabiting differences emerge among different types of unmet care needs (Supplementary Table 5). An additional analysis removing proxy respondents yielded results similar to the main results (Supplementary Table 6).

## Discussion

Partners are especially important in older adulthood in relation to care receipt (Lin, 2024), the need for which increases with age, with the onset of activity limitations (Freedman & Spillman, 2014). Given that cohabitation has become more prevalent among older adults over the past two decades, we seek to understand patterns of care and help received within these partnerships. We find little support for cohabitation as an incomplete institution among opposite-sex older adult couples and more support for cohabitation as an alternative to marriage, with one exception. For adverse consequences related to unmet care needs, cohabiting women have more unmet needs than married women, and the gender gap between men and women regarding unmet needs is larger among cohabiting than married couples.

Our overall results for cohabitation differ from those found by Noël-Miller (2011) who used data for adults aged 51 and older in need of care from 2000 to 2006, in that she found that cohabiting older adults were less likely to receive care than married older adults. We find similar rates of care between cohabiting and married older adults, largely consistent with a recent Dutch study that compared married, remarried, cohabiting, and living apart together (LAT) older adult relationships and reported few differences in receipt of personal care between these groups (van Groenou, 2019). Although we cannot sort out with the data at hand, it may be that as cohabitation becomes more commonplace, it becomes a more complete alternative to marriage, making earlier studies of cohabitors a more select group compared to contemporary cohabiting older adults. Alternatively, it may be that cohabitation is a more complete alternative to marriage in later life (relative to mid-life), when care needs increase.

Our study also diverged from Noël-Miller (2011) in terms of hours of assistance. We found that the average share of hours of assistance by the partner was similar across studies (84% our study, 83% Noël-Miller, 2011). Yet, in contrast to the larger share of overall care provided by cohabiting partners compared to married partners in Noël-Miller (2011), we found no difference in the share of hours by union status. However, we found that cohabiting older adults receive fewer hours of assistance from their partner relative to married older adults, in contrast to Noël-Miller (2011) who found no difference in hours. These contrasting findings may be due to differences in the data sources analyzed. Noël-Miller’s (2011) sample from the Health and Retirement Study (HRS) includes adults aged 51 and older, while the NHATS sample in this analysis includes adults aged 66 and older; in addition, the HRS data were from the early 2000s, while the NHATS data in this analysis span the 2010s into the 2020s. Care trends among partners may vary between middle and later life, with gender gaps closing among older adults compared to middle-aged adults (Glauber, 2017; Lima et al., 2008), and cohabitation rates have increased over time, especially among older adults.

Our finding that adverse consequences related to unmet care needs differ by union status and gender is new. We find that cohabitation is associated with similar rates of unmet needs (relative to both married men and women) for cohabiting men. However, cohabiting women in need of care are more likely to have unmet care needs than married women and cohabiting men, once adjusting for partnership and older adult characteristics. Given that cohabiting couples receive a majority (86%) of their total hours of assistance from a partner, these results suggest cohabitation functions as an alternative to marriage for men, but an incomplete institution for women regarding unmet care needs. These findings echo other studies showing that a partner is an important source of care, and potential for unmet care needs, in later life (Lin, 2024). Additional analysis (Supplementary Table 3) shows that cohabiting women had the highest rates of unmet care needs, especially mobility needs, which include getting in and out of bed, around inside one’s home, and getting outside. Mobility needs are the most common unmet care need among older adults (Freedman & Spillman, 2014), and these findings suggest that older cohabiting women may be at the greatest risk of unmet need for these tasks. Although models control for these characteristics, older cohabiting women have a lower physical capacity score on average, are more likely to have a partner who is older than them, and are less likely to talk to their partner about important matters. Taken together, these characteristics may put older cohabiting women at higher risk of unmet care needs.

We find a series of main gender effects that are noteworthy. Our results confirmed that women who are partnered to men are less likely to receive overall non-health-related help from their partners, as well as fewer overall hours and proportion from their partners, compared to men who are partnered to women. However, older women in need are more likely to receive health-related care from their partner than men. These findings are potentially explained by recent estimates that show that men are increasingly providing care and help to their partners (Cha & Ailshire, 2026), but mirror other work that shows that men are less likely to provide help to a partner (Allen, 1994; Dukhovnov & Zagheni, 2015; Glauber, 2017), especially with household tasks (Sheehan & Tucker-Drob, 2019). Taken together, the results suggest that gender norms may change in later adulthood or with younger cohorts of older men.

In additional analyses (Supplementary Table 4), even more evidence for the importance of investigating gender and care versus help task types emerged. While women are less likely than men to receive self-care care from their partner, they are more likely to receive both mobility and health-related household help. However, women are less likely to receive non-health-related household help from their partner than men but more likely to receive help with administrative tasks. Given prior work showing that men increasingly provide household help over time (Sheehan & Tucker-Drob, 2019), our results pinpoint the importance of understanding the differences between health-related household help and the division of labor. Although there are currently no universally standardized ways of distinguishing care from help in the literature (for a recent discussion, see Freedman & Wolff, 2020), the NHATS disablement framework aims to address these issues, which strengthens the ability of these data to address our research questions.

Our study has several limitations. First, our analysis focuses on individual reports rather than couples’ reports. Although nationally representative, this approach does not allow us to examine reciprocity between cohabiting and married couples, nor marital and relationship history. Second, we cannot account for selection into partnership type or partnership history due to a lack of measures, unobserved variable bias, or same-sex couples due to a small sample (Zhang et al., 2024). In addition, we cannot account for LAT couples, but a large majority (86%) of LAT older adult couples do not expect to either cohabit or marry their partner, so they are quite different (Wu & Brown, 2022). There may be additional nuances related to health and disability that we were unable to investigate here. For instance, women who experience disability may be at higher risk for partnership dissolution, but this may be buffered by relationship quality (Latham-Mintus et al., 2022), and care from a partner or spouse may decrease over time, partially due to the other partner’s own health declines (Swinkels et al., 2022). In addition, providing care to a partner may later affect the caregiving partner’s own health because even transitioning out of care later is not associated with health recovery (Uccheddu et al., 2019). More work could also be done to understand how caregiving duties an older adult already has influenced their partnership status, as studies show that women perceived men as less desirable if they felt they would increase the woman’s caregiving responsibilities (Harris, 2023). In addition, the complexity of living arrangements in later life (e.g., being in an assisted living facility) may complicate the story of whether they live apart by choice. Although we control for the number of biological and stepchildren, we did not explore the impact of other family members or caregivers, such as children-in-law, siblings, or grandchildren, which could influence gendered care patterns (Bertogg & Strauss, 2020; Cha & Ailshire, 2026). Finally, a small percentage of respondents’ answers are provided by a proxy, with two-thirds of those proxies being a partner. Prior to Round 12, proxies did not answer the social network item for respondents. We cannot assess whether there are biases introduced through the use of such proxies, but additional sensitivity analyses removing proxy respondents yield similar results to the current findings (see Supplementary Table 6).

In conclusion, our study investigates patterns of care received by older adults in married and cohabiting partnerships among a contemporary cohort of older adults in the United States. We find that late-life cohabitation is largely a complete institution similar to marriage for older men and women, with one key exception: older cohabiting women are more likely to have unmet care needs than married women and the gender gap is larger among cohabitors. The lack of clear expectations about care roles in cohabiting relationships, in addition to traditional gender role expectations and enactments, may put cohabiting women at greater risk of unmet care needs later in life. As cohabiting in later life becomes more common and the U.S. population continues to age, tracking trends in care and cohabitation will be an important undertaking.

## Supplementary Material

gbag136_Supplementary_Data

## Data Availability

Data are available on www.nhats.org. The study was not preregistered.
