# Supplementary material for: Cohabitation and unmet care needs in later life
Source: J Gerontol B Psychol Sci Soc Sci. 2026 Jul 11;81(10):gbag136. doi: 10.1093/geronb/gbag136 (PMC13553082; doi:10.1093/geronb/gbag136)
Supplement: Supplementary file 1 [file gbag136_supplementary_data.docx]

***The Journals of Gerontology, Series B: Psychological Sciences and Social Sciences* Supplementary Material: Patterson et al. Cohabitation and unmet care needs in later life.**

| Supplementary Table 1. Regression Coefficients for Adverse Consequences for Older Adults (Age 66 and Older) in Need of Care Who Co-reside in the Community | | | | |
| --- | --- | --- | --- | --- |
|  | Any adverse consequences (logit) | | | |
|  | Coeff. | SE | Coeff. | SE |
| Cohabiting (vs. Married) | 0.19 | (0.23) | -0.22 | (0.32) |
|  |  |  |  |  |
| Female (vs. Male) | -0.00 | (0.12) | -0.06 | (0.12) |
| x Cohabiting |  |  | 0.91* | (0.43) |
|  |  |  |  |  |
| **Partnership Characteristics** |  |  |  |  |
| Years married or living together | -0.00 | (0.00) | -0.00 | (0.00) |
| Partner is older | -0.17 | (0.12) | -0.15 | (0.12) |
| Partner needs help with personal care | 0.71*** | (0.15) | 0.72*** | (0.15) |
| Talk to partner about important matters | -0.17 | (0.12) | -0.17 | (0.12) |
| Total household income (logged) | 0.04 | (0.08) | 0.04 | (0.08) |
|  |  |  |  |  |
| **Older Adult Characteristics** |  |  |  |  |
| Physical Capacity Score | -0.27*** | (0.02) | -0.27*** | (0.02) |
| Dementia (Possible or Probable) | 0.29* | (0.12) | 0.29* | (0.12) |
| Number of children | 0.01 | (0.03) | 0.01 | (0.03) |
| Age (65-74 omitted) |  |  |  |  |
| 75 - 84 | -0.19 | (0.11) | -0.20 | (0.11) |
| 85+ | -0.35* | (0.15) | -0.36* | (0.15) |
| Race (White, non-Hispanic omitted) |  |  |  |  |
| Black, non-Hispanic | -0.43** | (0.14) | -0.43** | (0.14) |
| Hispanic | -0.27 | (0.17) | -0.27 | (0.17) |
| Other, non-Hispanic | -0.20 | (0.27) | -0.22 | (0.27) |
| College Degree | 0.48*** | (0.12) | 0.48*** | (0.12) |
| Has a Proxy Respondent | -0.25 | (0.20) | -0.26 | (0.20) |
|  |  |  |  |  |
| Survey Year (2012 omitted) |  |  |  |  |
| 2016 | 0.15 | (0.11) | 0.14 | (0.11) |
| 2023 | 0.25* | (0.11) | 0.25* | (0.11) |
|  |  |  |  |  |
| *Constant* | 0.69 | (0.85) | 0.73 | (0.85) |
| Note. Data are from the 2012, 2016, and 2023 National Health and Aging Trends Study (NHATS). Estimates are weighted and adjusted for survey design and repeated observations. N=4,283; 4,070 Married, 213 Cohabiting. *p<.05, **p<.01, ***p<.001 | | | | |

| Supplementary Table 2. Weighted Descriptive Statistics of Characteristics of Older Adults (Age 66 and Older) in Need of Care Who Coreside with Their Opposite-Sex Partner in the Community, by Sex, Mean (#) or Percentage (%) | | | | | | | | | | |
| --- | --- | --- | --- | --- | --- | --- | --- | --- | --- | --- |
|  | Older Men in Need of Care | | | |  | Older Women in Need of Care | | | | Sig. Diff. Between Cohabiting Men/Women |
|  | All | Married | Cohabiting | Sig. Diff. |  | All | Married | Cohabiting | Sig. Diff. |  |
| N | 2,468 | 2,341 | 127 |  |  | 1,814 | 1,729 | 86 |  |  |
| Weighted % of the sample |  | 94.5 | 5.5 |  |  |  | 95.5 | 4.5 |  |  |
|  |  |  |  |  |  |  |  |  |  |  |
| **Partnership Characteristics** |  |  |  |  |  |  |  |  |  |  |
| Years married or living together (#) | 40.6 | 41.9 | 18.3 | *** |  | 44.3 | 45.5 | 18.1 | *** |  |
| Partner is older (%) | 13.1 | 12.7 | 19.4 |  |  | 62.2 | 63.4 | 38.2 | ** | * |
| Partner needs help with personal care (%) | 10.1 | 10.1 | 9.6 |  |  | 8.7 | 8.9 | 5.5 |  |  |
| Talk to partner about important matters (%) | 83.6 | 83.8 | 79.7 |  |  | 67.6 | 68.0 | 60.4 |  | * |
| Total Household Income ($; in 2022 dollars) | 83,750 | 84,777 | 65,948 |  |  | 76,300 | 76,928 | 62,999 |  |  |
|  |  |  |  |  |  |  |  |  |  |  |
| **Older Adult Characteristics** |  |  |  |  |  |  |  |  |  |  |
| Physical Capacity Score (#) | 8.8 | 8.7 | 9.2 |  |  | 7.4 | 7.4 | 7.1 |  | ** |
| Dementia (Possible or Probable) (%) | 22.6 | 22.8 | 18.1 |  |  | 16.4 | 16.6 | 11.9 |  |  |
| Number of children (#) | 3.0 | 3.0 | 2.3 | ** |  | 2.9 | 2.9 | 2.3 | * |  |
| Age (%) |  |  |  |  |  |  |  |  |  |  |
| 65 - 74 | 47.7 | 46.9 | 61.6 | * |  | 54.5 | 54.7 | 49.2 |  |  |
| 75 - 84 | 40.4 | 41.0 | 29.8 |  |  | 38.2 | 38.1 | 40.6 |  |  |
| 85+ | 11.8 | 12.0 | 8.6 |  |  | 7.3 | 7.2 | 10.2 |  |  |
| Race/Ethnicity (%) |  |  |  |  |  |  |  |  |  |  |
| White, non-Hispanic | 81.3 | 81.6 | 75.2 |  |  | 79.8 | 80.3 | 68.9 |  |  |
| Black, non-Hispanic | 6.7 | 6.5 | 10.5 |  |  | 6.6 | 6.5 | 7.6 |  |  |
| Hispanic | 7.3 | 7.1 | 10.7 |  |  | 8.7 | 8.5 | 13.1 |  |  |
| Other, non-Hispanic | 4.7 | 4.8 | 3.6 |  |  | 5.0 | 4.7 | 10.3 |  |  |
| College Degree (%) | 34.4 | 35.1 | 22.2 | * |  | 24.1 | 24.3 | 21.3 |  |  |
| Has a proxy survey respondent (%) | 6.9 | 7.2 | 1.3 | *** |  | 4.7 | 4.9 | 1.5 | * |  |

| Supplementary Table 3. Weighted Descriptive Statistics of Help and Care Received by Older Adults (Age 66 and Older) in Need of Care Who Co-reside with Their Opposite-Sex Partner in the Community | | | | | | | | | |
| --- | --- | --- | --- | --- | --- | --- | --- | --- | --- |
|  | Men | | | |  | Women | | | |
|  | All | Married | Cohabiting | Sig. Diff. |  | All | Married | Cohabiting | Sig. Diff. |
| **Care from Partner** |  |  |  |  |  |  |  |  |  |
| Received care | 40.3 | 40.7 | 34.9 |  |  | 50.9 | 50.9 | 52.1 |  |
| *Self-Care* | 27.5 | 28.0 | 19.0 |  |  | 23.9 | 24.0 | 22.2 |  |
| *Mobility* | 14.8 | 15.1 | 8.4 |  |  | 24.3 | 24.1 | 28.6 |  |
| *Household help* | 25.8 | 25.8 | 25.4 |  |  | 37.2 | 37.3 | 35.1 |  |
| **Help from Partner** |  |  |  |  |  |  |  |  |  |
| Received help | 93.6 | 93.7 | 93.0 |  |  | 83.4 | 83.4 | 82.1 |  |
| *Household help* | 85.5 | 85.5 | 85.5 |  |  | 64.1 | 64.6 | 53.9 |  |
| *Administrative tasks* | 63.8 | 64.1 | 60.0 |  |  | 69.0 | 69.3 | 64.0 |  |
|  |  |  |  |  |  |  |  |  |  |
| **Hours of Care/Help Received from Partner (if any received from partner)** | | | | | | | | | |
| *Partner hours* | 112.7 | 114.5 | 80.6 | ** |  | 73.4 | 74.4 | 52.39 |  |
| *Total hours (from anyone)* | 130.5 | 132.8 | 91.69 | *** |  | 98.31 | 99.46 | 74.22 |  |
| *Percentage of hours received from partner* | 87.8 | 87.7 | 89.7 |  |  | 79.6 | 79.6 | 79.0 |  |
|  |  |  |  |  |  |  |  |  |  |
| **Adverse Consequences Related to Unmet Care Needs** | | | |  |  |  |  |  |  |
| Adverse consequence for any activity | 26.0 | 26.3 | 21.1 |  |  | 30.1 | 29.3 | 45.4 | * |
| *Self-Care* | 9.7 | 10.0 | 4.7 | * |  | 11.6 | 11.3 | 17.9 |  |
| *Mobility* | 13.5 | 13.6 | 12.1 |  |  | 18.8 | 18.2 | 31.1 | * |
| *Household help* | 11.7 | 11.8 | 9.6 |  |  | 12.3 | 12.2 | 14.5 |  |
| Note. Data are from the 2012, 2016, and 2023 National Health and Aging Trends Study (NHATS). Estimates are weighted and adjusted for survey design and repeated observations. Sig. Diff. = significant difference between married and cohabiting older adults among men / among women.  *p<.05, **p<.01, ***p<.001 | | | | | | | | | |

| Supplementary Table 4. Regression Coefficients for Each Individual Types of Care or Help | | | | |
| --- | --- | --- | --- | --- |
|  | Received Self-Care Care | | | |
|  | Coeff. | SE | Coeff. | SE |
| Cohabiting (vs. Married) | -0.27 | (0.25) | -0.34 | (0.33) |
| Female (vs. Male) | -0.51*** | (0.12) | -0.52*** | (0.13) |
| x Cohabiting |  |  | 0.18 | (0.48) |
| *Constant* | -0.13 | (0.83) | -0.13 | (0.83) |
|  | Received Mobility Care | | | |
|  | Coeff. | SE | Coeff. | SE |
| Cohabiting (vs. Married) | -0.15 | (0.30) | -0.47 | (0.50) |
| Female (vs. Male) | 0.47*** | (0.14) | 0.44** | (0.14) |
| x Cohabiting |  |  | 0.57 | (0.59) |
| *Constant* | -0.52 | (0.98) | -0.50 | (0.97) |
|  | Received Household Care | | | |
|  | Coeff. | SE | Coeff. | SE |
| Cohabiting (vs. Married) | 0.02 | (0.26) | 0.21 | (0.31) |
| Female (vs. Male) | 0.43*** | (0.12) | 0.46*** | (0.12) |
| x Cohabiting |  |  | -0.44 | (0.48) |
| *Constant* | 1.40 | (0.84) | 1.39 | (0.84) |
|  | Received Household Help | | | |
|  | Coeff. | SE | Coeff. | SE |
| Cohabiting (vs. Married) | -0.28 | (0.29) | -0.06 | (0.36) |
| Female (vs. Male) | -1.22*** | (0.12) | -1.19*** | (0.12) |
| x Cohabiting |  |  | -0.41 | (0.52) |
| *Constant* | 0.85 | (0.92) | 0.82 | (0.92) |
|  | Received Administrative Help | | | |
|  | Coeff. | SE | Coeff. | SE |
| Cohabiting (vs. Married) | -0.08 | (0.21) | -0.00 | (0.26) |
| Female (vs. Male) | 0.34** | (0.11) | 0.35** | (0.11) |
| x Cohabiting |  |  | -0.20 | (0.40) |
| *Constant* | 0.43 | (0.75) | 0.43 | (0.75) |
| Note. Data are from the 2012, 2016, and 2023 National Health and Aging Trends Study (NHATS). Estimates are weighted and adjusted for survey design and repeated observations. Models control for all variables in the main models.  *p<.05, **p<.01, ***p<.001. | | | | |

| Supplementary Table 5. Regression Coefficients for Each Individual Group of Unmet Need Type | | | | |
| --- | --- | --- | --- | --- |
|  | Unmet Personal Care | | | |
|  | Coeff. | SE | Coeff. | SE |
| Cohabiting (vs. Married) | 0.01 | (0.31) | -0.60 | (0.50) |
| Female (vs. Male) | -0.12 | (0.18) | -0.18 | (0.19) |
| x Cohabiting |  |  | 1.11 | (0.62) |
| *Constant* | 0.39 | (1.16) | 0.41 | (1.15) |
|  | Unmet Mobility Care | | | |
|  | Coeff. | SE | Coeff. | SE |
| Cohabiting (vs. Married) | 0.28 | (0.27) | -0.04 | (0.41) |
| Female (vs. Male) | 0.15 | (0.15) | 0.11 | (0.16) |
| x Cohabiting |  |  | 0.63 | (0.51) |
| *Constant* | -0.00 | (1.04) | 0.02 | (1.04) |
|  | Unmet Household Care | | | |
|  | Coeff. | SE | Coeff. | SE |
| Cohabiting (vs. Married) | -0.19 | (0.30) | -0.34 | (0.40) |
| Female (vs. Male) | 0.06 | (0.15) | 0.04 | (0.16) |
| x Cohabiting |  |  | 0.33 | (0.54) |
| *Constant* | -0.60 | (1.08) | -0.59 | (1.08) |
| Note. Data are from the 2012, 2016, and 2023 National Health and Aging Trends Study (NHATS). Estimates are weighted and adjusted for survey design and repeated observations. No significant differences to report for this table. Models control for all variables in the main models. | | | | |

| Supplementary Table 6. Regression Coefficients for All Outcomes for Older Adults (Age 66 and Older) in Need of Care Who Co-reside in the Community, without Proxy Respondents | | | | |
| --- | --- | --- | --- | --- |
|  | Received care from partner (logit) | | | |
|  | Coeff. | SE | Coeff. | SE |
| Cohabiting (vs. Married) | -0.07 | (0.23) | -0.10 | (0.28) |
| Female (vs. Male) | 0.28* | (0.12) | 0.28* | (0.12) |
| x Cohabiting |  |  | 0.07 | (0.44) |
| *Constant* | 2.34** | (0.82) | 2.35** | (0.82) |
|  | Received help from partner (logit) | | | |
|  | Coeff. | SE | Coeff. | SE |
| Cohabiting (vs. Married) | -0.03 | (0.32) | -0.08 | (0.42) |
| Female (vs. Male) | -1.03*** | (0.17) | -1.04*** | (0.17) |
| x Cohabiting |  |  | 0.08 | (0.57) |
| *Constant* | 1.51 | (1.31) | 1.52 | (1.31) |
|  | Total hours of assistance from partner, if any (OLS) | | | |
|  | Coeff. | SE | Coeff. | SE |
| Cohabiting (vs. Married) | -21.40* | (9.55) | -22.89* | (10.95) |
| Female (vs. Male) | -52.33*** | (6.22) | -52.58*** | (6.48) |
| x Cohabiting |  |  | 3.96 | (16.41) |
| *Constant* | 236.97*** | (45.92) | 237.01*** | (45.91) |
|  | Percent of hours of assistance from partner, if any (OLS) | | | |
|  | Coeff. | SE | Coeff. | SE |
| Cohabiting (vs. Married) | 0.01 |  | 0.02 | (0.03) |
| Female (vs. Male) | -0.07*** | (0.01) | -0.07*** | (0.01) |
| x Cohabiting |  |  | -0.03 | (0.04) |
| *Constant* | 0.86*** | (0.09) | 0.86*** | (0.09) |
|  | Any adverse consequences (logit) | | | |
|  | Coeff. | SE | Coeff. | SE |
| Cohabiting (vs. Married) | 0.15 |  | -0.26 | (0.33) |
| Female (vs. Male) | -0.07 | (0.13) | -0.14 | (0.13) |
| x Cohabiting |  |  | 0.94* | (0.43) |
| *Constant* | 0.92 | (0.89) | 0.96 | (0.89) |
| Note. Data are from the 2012, 2016, and 2023 National Health and Aging Trends Study (NHATS). Estimates are weighted and adjusted for survey design and repeated observations. N=3,948; 3,744 Married, 204 Cohabiting. Models control for all variables in main models.  *p<.05, **p<.01, ***p<.001 | | | | |
